# Sonographic Lobe Localization of Alveolar-Interstitial Syndrome in the Critically Ill

**DOI:** 10.1155/2012/179719

**Published:** 2012-05-09

**Authors:** Konstantinos Stefanidis, Stavros Dimopoulos, Chrysafoula Kolofousi, Demosthenes D. Cokkinos, Katerina Chatzimichail, Lewis A. Eisen, Mitchell Wachtel, Dimitrios Karakitsos, Serafim Nanas

**Affiliations:** ^1^Department of Radiology, Evangelismos Hospital, NKUA, 10676 Athens, Greece; ^2^1st Critical Care Medicine Department, Evangelismos Hospital, NKUA, 10676 Athens, Greece; ^3^Radiology Department, Attikon University Hospital, 12462 Athens, Greece; ^4^Division of Critical Care Medicine, Department of Medicine, Jay B. Langner Critical Care Service Montefiore Medical Center, Albert Einstein College of Medicine, 10467 Bronx NY, USA; ^5^Department of Biostatistics, Texas Tech University, 79409 Lubbock, TX, USA; ^6^Intensive Care Unit, General State Hospital of Athens, 11523 Athens, Greece

## Abstract

*Introduction*. Fast and accurate diagnosis of alveolar-interstitial syndrome is of major importance in the critically ill. We evaluated the utility of lung ultrasound (US) in detecting and localizing alveolar-interstitial syndrome in respective pulmonary lobes as compared to computed tomography scans (CT). *Methods*. One hundred and seven critically ill patients participated in the study. The presence of diffuse comet-tail artifacts was considered a sign of alveolar-interstitial syndrome. We designated lobar reflections along intercostal spaces and surface lines by means of sonoanatomy in an effort to accurately localize lung pathology. Each sonographic finding was thereafter grouped into the respective lobe. *Results*. From 107 patients, 77 were finally included in the analysis (42 males with mean age = 61 ± 17 years, APACHE II score = 17.6 ± 6.4, and lung injury score = 1.0 ± 0.7). US exhibited high sensitivity and specificity values (ranging from over 80% for the lower lung fields up to over 90% for the upper lung fields) and considerable consistency in the diagnosis and localization of alveolar-interstitial syndrome. *Conclusions*. US is a reliable, bedside method for accurate detection and localization of alveolar-interstitial syndrome in the critically ill.

## 1. Introduction

Pulmonary diseases with involvement of the alveolar space and the interstitium (alveolar-interstitial syndrome) are common in the critically ill. Diagnostic assessment of the alveolar-interstitial syndrome includes chest radiography and computed tomography (CT). Chest CT is considered the “gold standard” test for the diagnosis of most pulmonary disorders in the intensive care unit (ICU). However, serial CT examinations may be required to followup the clinical course of pulmonary disorders and the results of therapy increasing radiation exposure. Also, this may be time consuming and hazardous as critically ill patients who oftentimes suffer from severe respiratory insufficiency are transferred to another unit.

Historically, lung was considered a poorly accessible organ for ultrasound (US) assessment mainly due to abundance of air. However, in patients with lung disease extending to the pleura, US can be particularly useful for a wide range of applications [1, 2]. Recent studies have shown the significant role of lung US in detecting pulmonary diseases [3–15]. Areas of ground-glass adjacent to the pleura, areas of consolidation and areas of thickening of the interstitium can be easily detected using lung US [3–13]. The sonographic imaging of pulmonary diseases is based on the detection and quantification of “comet-tails” lines known as “B-lines” or lung rockets [5], generated by reverberation of the US beam. Previous studies have shown that the presence of multiple lines perpendicular to the pleura with a distance of 3 mm or less and a distance of 7 mm and more are representative of ground-glass areas and of subpleura interlobular septa thickening, respectively [3–5]. Although there have been several studies reporting the possible role of lung US in detecting the alveolar-interstitial syndrome [3–13], its application in routine ICU practice remains unclear. 

The aim of this study was to investigate the utility of a simple lung US protocol in detecting and localizing areas of alveolar and/or interstitial involvement in respective pulmonary lobes as compared to thoracic CT scans in critical care patients.

## 2. Materials and Methods

### 2.1. Study Population

We enrolled 107 consecutive patients with respiratory failure necessitating mechanical ventilation who were admitted to our medical ICU during a 12-month period. Patients with an ICU stay longer than 48 hours who underwent chest CT for diagnostic purposes were included in this study. Patients with pneumothorax, subcutaneous emphysema, mesothelioma, massive effusion, pneumonectomy, and body mass index (BMI) ≥40 kg/m^2^ (class III obesity) were excluded. All patients were sedated under mechanical ventilation set at the volume assist-control mode. Informed consent was obtained from all patients or their relatives and the study was approved by institutional ethics committee.

### 2.2. Study Protocol

**Figure 1:**
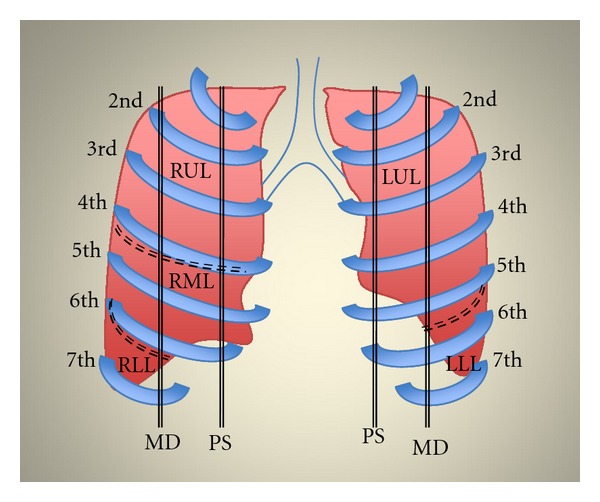
Anterior view of the lung. Schematic representation of pulmonary lobes in relation to ribs and intercostal spaces along parasternal (PS) and midclavicular (MD) lines, respectively. Dashed lines correspond to major and minor lung fissures (RUL: right upper lobe; RML: right mid lobe, RLL: right lower lobe; LUL: left upper lobe; LLL: left lower lobe).

**Figure 2:**
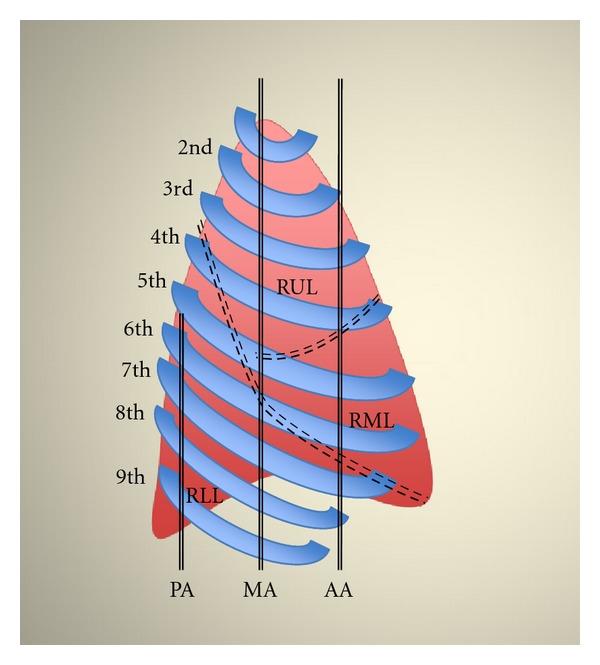
Lateral view of the right lung. Schematic representation of pulmonary lobes in relation to ribs and intercostal spaces along anterior axillary (AA), midaxillary (MD), and posterior axillary (PA) lines, respectively.

**Figure 3:**
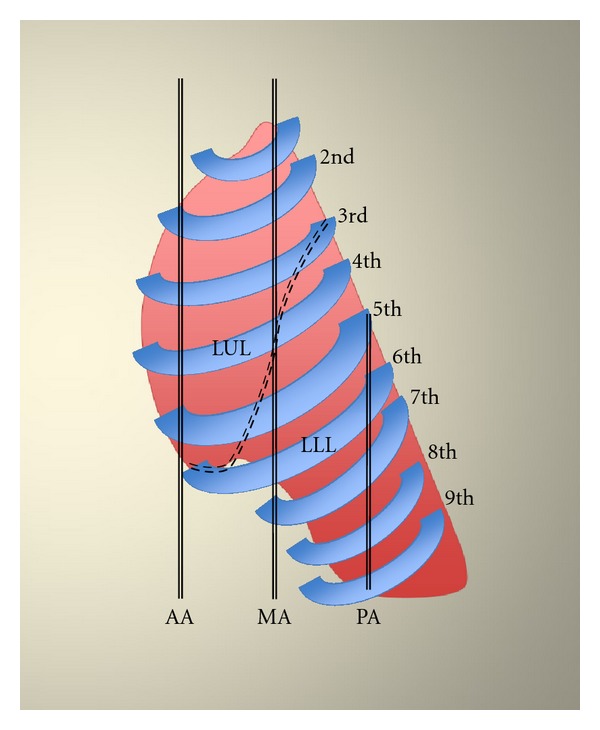
Lateral view of the left lung. Schematic representation of pulmonary lobes in relation to ribs and intercostal spaces along anterior axillary (AA), midaxillary (MD), and posterior axillary (PA) lines, respectively.

Lung US was performed before CT scan, within an interval of 30 min, by an independent expert radiologist who was blinded to the subjects' identity and to the CT results. The portable US system Vivid 7 (GE, Wauwatosa, WI, USA) equipped with a sector array probe (1.5–3.8 MHz) was utilized. All patients were examined in supine or semirecumbent position. US examinations consisted of bilateral scanning of the anterior and lateral chest of the right and left hemithorax. Lung US was performed from the second to the fifth intercostal space from parasternal to midaxillary line, for the right lung; from the second to the fourth intercostal space from parasternal to midaxillary line, for the left lung, respectively (Figures 1, 2, and 3). This also included sonographic depiction of the fissures. Along the posterior axillary line, scanning was performed at the level of seventh and eighth intercostal space. Notably, examination of the left fifth intercostal space was not performed since the heart blocks the visibility of the wall interface. All patients were examined in end-expiration to avoid displacements of the lower borders of the lung. The intercostal spaces which were scanned along the lines were grouped into respective pulmonary lobes (Table 1). Results of US scanning in each pulmonary lobe were recorded and compared with CT findings in the same lobe. Presence of A-lines was considered normal [3]. Alveolar-interstitial syndrome in each lobe was defined as the presence of more than two comet-tail artifacts perpendicular to the pleural line [3–5]. Alveolar pattern included also pleural-based consolidations described sonographically as heterogeneous tissue-like patterns resembling the echogenicity of the liver with hyperechoic punctiform or linear artifacts, corresponding to air bronchograms [3–5]. 

Thoracic CT scans were performed from the apex to the diaphragm using a Tomoscan (GE, WI, USA). All images were observed and photographed at a window width of 1,600 HU and a level of −600 HU. An independent radiologist, who was blinded to subjects' identity and to lung US results, was assigned to interpret the CT results. All findings were recorded and assigned to the appropriate pulmonary lobe. Alveolar-interstitial syndrome was defined according to the Fleischner Society's recommendations [16] as the presence of one or the combination of ground-glass opacities, consolidation, reticulation, and septal thickening.

### 2.3. Statistical Analysis

Continuous variables are presented as mean ± standard deviation (SD). The accuracy of lung US in detecting alveolar-interstitial syndrome was evaluated by means of sensitivity = (true positive/(true positive + false negative)); specificity = (true negative/(true negative + false positive)); positive predictive value = (true positive/(true positive + false positive)); negative predictive value = (true negative/(true negative + false negative)); and diagnostic accuracy = (true positive + true negative)/(true positive + true negative + false positive + false negative). Cohen's weighted kappa was calculated to express the degree of agreement between lung US and thoracic CT scan in diagnosing and localizing the alveolar-interstitial syndrome in all respective pulmonary lobes [17], while 2.5th and 97.5th percentiles of 5,000 bootstrap replicates estimated 95% confidence intervals. The bootstrap is a resampling method used for estimating a distribution, from which various measures of interest can be calculated [18, 19]. A *P*-value (two-sided in all tests) of <0.05 was considered significant. Analysis was performed with the R2.10.1 statistical package (R Development Core Team, 2009. R: A language and environment for statistical computing. R Foundation for Statistical Computing, Vienna, Austria).

## 3. Results

From 107 consecutive patients studied, 77 were finally enrolled (42 males with mean age = 61 ± 17 years, acute physiology and chronic health evaluation score (APACHE) II = 17.6 ± 6.4, and lung injury score = 1.0 ± 0.7). Thirty patients were excluded from the study. The causes were an ICU stay less than 48 hours (*n* = 18), subcutaneous emphysema (*n* = 8), pneumonectomy (*n* = 2), and a BMI ≥40 (*n* = 2). Various causes of admission in the ICU were recorded such as multiple organ dysfunction syndrome (*n* = 23), trauma (*n* = 17), postsurgical complications (*n* = 15), exacerbation of chronic obstructive pulmonary disease (COPD, *n* = 6), and miscellaneous (*n* = 16).

**Figure 4:**
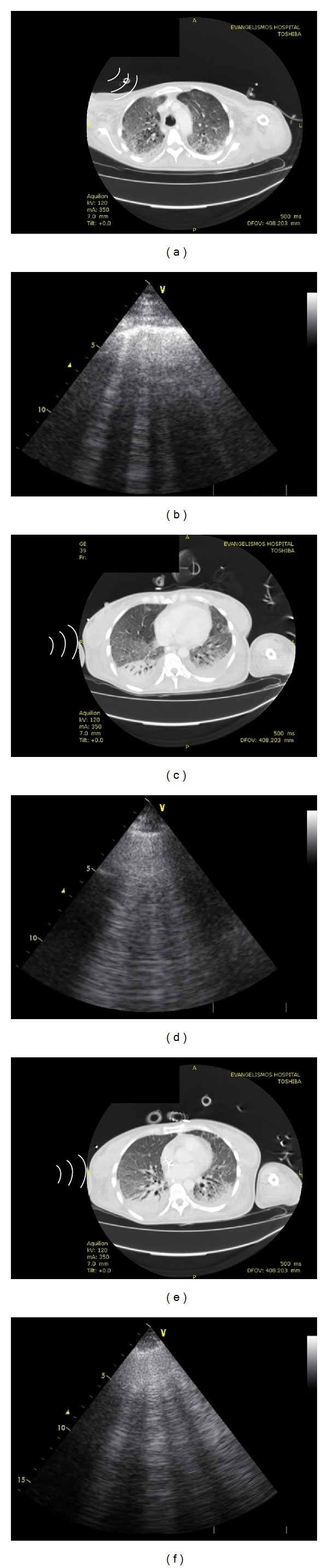
Computed tomography (CT) scans showing areas of “ground glass” opacification and bilateral-dependent areas of dense consolidation in a patient with acute respiratory distress syndrome (right panel). Lung ultrasound scans in the same patient depicting B-lines arising from the pleural line, confirming thus a pattern of diffuse alveolar-interstitial syndrome (left panel).

Hence, a total of 144 hemithoraces were evaluated both by US and CT scans according to the study protocol (Figure 4). Alveolar-interstitial syndrome was diagnosed by CT scans in 42/77 (54%), 49/77 (64%) and 61/77 (79%) patients for the upper, mid-, and lower right lobes, respectively. In the left lung, alveolar-interstitial syndrome was diagnosed by CT scans in 38/77 (49%) and 65/77 (84%) patients for the upper and the lower lobe, respectively. US detected alveolar-interstitial syndrome in 39/77 (50%), 47/77 (61%) and 50/77 (65%) patients for the upper, mid-, and lower right lobes, respectively. In the left lung, sonographic alveolar-interstitial syndrome was detected in 36/77 (47%) and 56/77 (73%) patients for the upper and the lower lobe, respectively. Diagnostic accuracy of lung US in detecting alveolar-interstitial syndrome is presented on Table 2.

**Figure 5:**
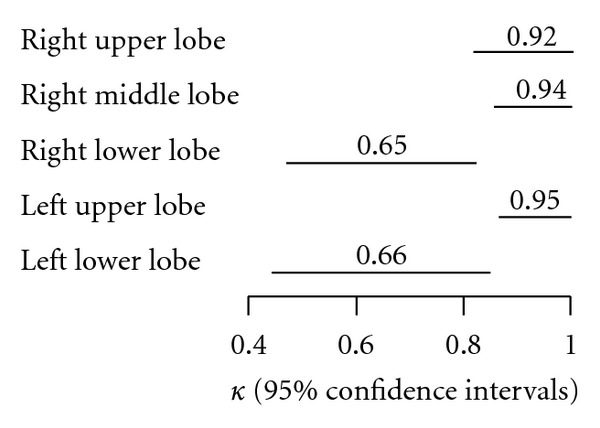
Cohen's kappa values by lobe of lung, with lines displaying bootstrap 95% confidence intervals.

Agreement between lung US and CT scans was evaluated according to kappa values and 95% confidence intervals were calculated by bootstrap analysis, for all respective pulmonary lobes: right upper 0.92 (0.82–1.00), mid 0.94 (0.86–1.00), lower 0.65 (0.47–0.82); left upper 0.95 (0.87–1.00), lower 0.66 (0.45–0.85), respectively (all *P* < 0.01) (Figure 5).The overall agreement, involving all lung fields bilaterally, between US and CT in the diagnosis and appropriate lobe localization of the alveolar-interstitial syndrome, was substantial: 0.78 (0.66–0.89; *P* < 0.01).

## 4. Discussion

In this study, lung US showed high sensitivity and specificity in the detection of alveolar-interstitial syndrome. The present results revealed substantial agreement between lung US and CT scans in detecting alveolar-interstitial syndrome in critical care patients.

The comet-tail artifact, a form of reverberation of echoes, was first described by Ziskin [20]. Since then, this repetition artifact noted at the lung surfaces in normal and pathologic clinical conditions [9], was sonographically correlated with the detection of alveolar-interstitial syndrome [3–8]. In previous reports studying the alveolar-interstitial syndrome, the chest wall was divided into anterior and lateral chest wall or into four areas divided for each hemithorax, two anterior, (upper and lower) and two lateral, (upper and basal) [3–5]. We designated lobar reflections along intercostal spaces and surface lines by means of sonoanatomy in an effort to accurately localize lung disease. Our results confirmed high sensitivity and specificity of lung US in diagnosing alveolar-interstitial syndrome as others have previously reported [3]. In patients with acute respiratory distress syndrome (ARDS) the presence of B-lines yielded an accuracy of 97% in the diagnosis of alveolar-interstitial syndrome [5]. B-lines were correlated with subpleural interstitial oedema and were suggested as potential non-invasive measures of pulmonary artery occlusion pressure in the critically ill [21]. In addition, two US studies that investigated the detection of alveolar-interstitial syndrome for the diagnosis of lung contusion presented 94% and 86% sensitivity and 96% and 97% specificity, respectively [12, 13]. In a recent study of 42 critical care patients, lung US presented a sensitivity, specificity and diagnostic accuracy of 94%, 93%, and 94% for detecting interstitial syndrome, respectively [7]. The efficacy of lung US in detecting areas of consolidations has been reported in previous studies [7–10]. Also, B-lines were used in the differential diagnosis between acute cardiogenic pulmonary oedema and ARDS, acute pulmonary oedema and exacerbation of COPD and in dyspnoea diagnostic protocols such as the Blue Protocol [22–24]. Interpretation of lung US artifacts can be helpful in various clinical scenarios (i.e., presence of comet-tails artifacts excludes the existence of pneumothorax) [14]. Study of these artifacts according to several research groups, allows evaluation of lung aeration in patients with ARDS [25, 26]. The association of B-lines with the presence of extravascular lung water [27, 28] may extend the role of lung US in assessing lung aeration. B-lines have been studied in cardiogenic and high-altitude pulmonary oedema [29, 30], following medical treatment of patients with acute decompensated heart failure [31], in patients undergoing hemodialysis [32], and in patients with community-acquired and ventilator-associated pneumonia [33]. Taking into account all previous reports, the present results reinforce the significance of lung US utility in the diagnosis of alveolar and interstitial pathology. Additionally, we were able to localize pulmonary disease of the alveolar space and/or the interstitium to respective lobes. A simple reproducible protocol had good diagnostic accuracy compared to the gold standard of CT scan. Localization to particular pulmonary lobes could be useful to aid in the differential diagnosis of respiratory disease. Moreover, a bedside test that can localize pulmonary disease could potentially be useful to guide diagnostic procedures such as bronchoscopy.

**Limitaions**

This study has several limitations. Lung US was performed on the anterior and lateral chest areas and not on dorsal areas to avoid displacement of patients. This might have increased the false negative cases especially for posterior disease processes. Indeed, our results revealed lower sensitivity and specificity values for lung US in the lower pulmonary lobes and decreased extent of agreement with CT scan findings in these areas. Dorsal scans could have improved the efficacy of lung US in detecting areas of ground-glass, consolidation, and areas of interstitial involvement in the posterior lung. If clinically warranted and attention is paid to patient safety, a more complete exam could be performed which included the dorsum though the accuracy of such an exam is unknown. Another issue that could explain imperfect diagnostic accuracy is the limited capability of US to detect pulmonary pathology that does not reach the pleura [34]. However, alveolar-interstitial syndrome is generally extended to the lung periphery. Our designated sonoanatomical correspondence of the intercostal spaces with the appropriate pulmonary lobe also represents a methodology limitation. The somatotype of the patient and underlying pulmonary pathology such as atelectasis and diaphragm paralysis may alter the anatomical correspondence of the pulmonary lobes with the intercostal spaces [27]. The performance in patients with anatomic variants such as accessory fissures is unknown. Since obese patients were excluded, results cannot be extrapolated to such patients. Finally, in this study, a single experienced observer performed all the US examinations to reduce bias. Lung US is considered an operator dependent test; however, high degree of inter- and intraobserver reproducibility has been previously reported for several indications [3, 4].

Despite the aforementioned limitations, this study demonstrated a high accuracy of lung US in diagnosing the alveolar-interstitial syndrome in the ICU. US exhibited substantial agreement with thoracic CT and showed consistency in the localization of lung pathology to respective lobes, even if the dorsal areas of the lung were not scanned. Sonography can be easily performed at the bedside, free of radiation exposure; hence, it may represent a promising alternative to CT in the monitoring of pulmonary disorders [35]. In conclusion, we provided evidence that lung US represents a reliable and accurate bedside test for assessing and localizing pulmonary disease of the interstitium and/or the alveolar space in critical care patients.

## Figures and Tables

**Table 1:** Ultrasound scanned intercostal spaces grouped in respective pulmonary lobes.

	PS	MDC	AA	MA	PA
Right lung					

RUL	2nd, 3rd LIS	2nd, 3rd LIS	2nd, 3rd LIS	2nd, 3rd, 4th LIS	—
RML	4th, 5th LIS	4th, 5th LIS	4th, 5th LIS	5th LIS	—
RLL	—	—	—	—	7th, 8th LIS

Left lung					

LUL	2nd, 3rd, 4th LIS	2nd, 3rd, 4th LIS	2nd, 3rd, 4th LIS	2nd, 3rd LIS	—
LLL	—	—	—	4th LIS	7th, 8th LIS

RUL: right upper lobe, RML: right mid lobe, RLL: right lower lobe; LUL: left upper lobe, LLL: left lower lobe, PS: parasternal line, MDC: midclavicular line, AA: anterior axillary line, MA: mid axillary line, PA: posterior axillary line, LIS: lung intercostal space.

**Table 2:** Accuracy of lung ultrasound in diagnosing alveolar-interstitial syndrome in respective pulmonary lobes.

	Sensitivity (%)	Specificity (%)	PPV (%)	NPV (%)	DA (%)
RUL	93	91	83	91	92
RML	96	96	98	93	96
RLL	82	87	96	56	83
LUL	95	87	88	94	91
LLL	86	92	98	55	87

PPV: positive predictive value; NPV: negative predictive value; DA: diagnostic accuracy; RUL: right upper lobe; RML: right mid lobe, RLL: right lower lobe; LUL: left upper lobe; LLL: left lower lobe.
